# Mebendazole Reduces Vascular Smooth Muscle Cell Proliferation and Neointimal Formation Following Vascular Injury in Mice

**DOI:** 10.1371/journal.pone.0090146

**Published:** 2014-02-27

**Authors:** Jintao Wang, Hui Wang, Chiao Guo, Wei Luo, Alyssa Lawler, Aswin Reddy, Julia Wang, Eddy B. Sun, Daniel T. Eitzman

**Affiliations:** University of Michigan, Department of Internal Medicine, Cardiovascular Research Center, Ann Arbor, Michigan, United States of America; William Harvey Research Institute, Barts and The London School of Medicine and Dentistry, Queen Mary University of London, United Kingdom

## Abstract

Mebendazole is an antihelminthic drug that exerts its effects via interference with microtubule function in parasites. To determine the utility of mebendazole as a potential treatment for vascular diseases involving proliferation of vascular smooth muscle cells, the effects of mebendazole on vascular smooth muscle cell proliferation were tested *in vitro* and in a mouse model of arterial injury. *In vitro,* mebendazole inhibited proliferation and migration of murine vascular smooth muscle cells and this was associated with altered intracellular microtubule organization. To determine *in vivo* effects of mebendazole following vascular injury, femoral arterial wire injury was induced in wild-type mice treated with either mebendazole or placebo control. Compared with placebo-treated mice, mebendazole-treated mice formed less neointima at the site of injury. Mebendazole is effective at inhibiting vascular smooth muscle cell proliferation and migration, and neointimal formation following arterial injury in mice.

## Introduction

Vascular smooth muscle cells (VSMC) play a prominent role in many vascular diseases [1]–[3]. In response to arterial injury, vascular smooth muscle cells respond by transforming from a quiescent, contractile phenotype to a proliferative, migratory, synthetic phenotype [1], [2]. Through proliferation, migration and production of extracellular matrix, VSMCs contribute to the obstructive vascular lesions observed in atherosclerosis as well as in restenosis following stenting [3]. In the case of restenosis following stenting, drug-eluting stents have been successful in limiting restenosis, however because of the high numbers of stents deployed each year, restenosis and recurrent restenosis are still commonly encountered. Safe and effective options to treat recurrent restenosis are still needed [4], [5].

Mebendazole (MZ) is a benzimidazole drug used as an antihelminthic agent [6]. Its anti-parasitic effect is presumably due to disruption of microtubule function in parasite cells [7], [8]. Because drugs that impair microtubule function may be useful in limiting restenosis [9], we tested the capacity of MZ to inhibit proliferation of VSMCs *in vitro* and *in vivo*.

## Materials and Methods

### Ethics Statement

All procedures complied with the Principles of Laboratory and Animal Care established by the National Society for Medical Research and were approved by the University of Michigan Committee on Use and Care of Animals.

### Animals

Wild-type C57BL6/J male mice were purchased from the Jackson Laboratory (Bar Harbor, ME). Male 8 week old mice were fed a standard laboratory rodent diet (#5001, TestDiet, Richmond, IN) in specific pathogen-free facilities.

### Cell Culture

Murine aorta smooth muscle cells (MOVAS, CRL-2797™, ATCC) were grown in Dulbecco modified Eagle medium (DMEM, Gibco Inc) containing 10% fetal bovine serum (FBS, Gibco Inc) and 0.2 mg/ml G-418, and passaged 2 to 3 times before use in assays.

### Cell Proliferation Assay

MOVAS cells (1×10^4^/well) were seeded in 96-well plates (Cat# 3300, Corning Inc) (three wells for each group). DMSO or MZ of indicated concentration (Sigma, St. Louis, MO) were added. Forty-eight hours later, cell proliferation was measured with the XTT Cell Proliferation Assay Kit (ATCC) according to manufacturer’s instructions and expressed as percentage of the DMSO-treated control group. To inhibit cell apoptosis induced with MZ, cells were treated with 50 µM Caspase-3/7 Inhibitor I (Santa Cruz Biotechnology, Inc., TX).

### Cell Wound Healing Assay

MOVAS were seeded in 6-well plates (Cat# 3516, Corning Inc) and allowed to grow to form a confluent monolayer. The monolayer was scratched using a sterile 200 µl pipette tip and washed twice with PBS to remove cell debris. The wound area was photographed with a phase contrast microscope and boundaries were marked (3 different fields per well and three wells for each group). Wound areas were measured with ImageJ software (NIH, Bethesda, MD) and recovered surface area over 24 hours was calculated and compared between MZ-treated and control wells [10].

To study the effect of MZ on cell apoptosis, active caspase 3/7 positive cells were stained. Three wells for each group and three fields of each well were imaged.

To study the effect of MZ on migrated cells, MOVAS cells were treated with 1 µM MZ 24 hours after scratch and observed for another 24 hours.

To inhibit cell apoptosis induced with MZ in this assay, cells were treated with 50 µM Caspase-3/7 Inhibitor I (Santa Cruz Biotechnology, Inc., TX).

### Transwell Migration Assay

MOVAS cells cultured to 80% confluence were starved in serum free medium for 24 hours before the assay. The inserts (Cat # 07-200-150, Corning Inc.) were rehydrated by adding 100 µL of warm serum free media to each insert and 500 µL to each receiver well (three inserts for each group). The inserts were incubated at 37°C in a CO_2_ incubator for 1 hour before the assay. The starved cells were harvested in serum free medium and adjusted to 1×10^6^ cells/ml and DMSO or 1 µM MZ was added. 100 µl cells were added to each insert. 650 µl of serum free medium with DMSO or 1 µM MZ was added to the receiver well. The transwell plate was then incubated at 37°C in a CO_2_ incubator for another 18 hours. Cells from unmigrated (top) side were removed with Q-tip/cotton swab. Migrated cells were then fixed by submerging inserts in 10% formalin for 10 minutes and cold methanol for another 5 minutes. Inserts were washed with PBS once and cells were stained with Hematoxylin. Migrated cells were counted with microscope under high power (40X). Three high power fields were counted for each insert.

### Femoral Artery Injury

Mouse femoral artery injury was performed using a model of wire-induced vascular injury as described previously [11]. This model causes endothelial damage at the site of injury followed by intimal hyperplasia. Mice were anesthetized with intraperitoneal sodium pentobarbital (120 mg/kg; Butler Co), then secured in the supine position and placed under a dissecting microscope (Nikon SMZ-2T, Mager Scientific Inc). After a midline incision was made on the ventral left thigh region, the femoral nerve was isolated and pulled aside. The distal and proximal ends of the femoral artery were held with surgical suture for temporary control of blood flow. A small arterial branch superior to the muscular branch of the femoral artery was cauterized (DELC Cauterizer, Aaron Medical Industries Inc). One drop of 1% lidocaine was used to prevent arterial spasm. An arteriotomy of the femoral artery muscular branch was then performed. A straight guide wire (0.38 mm diameter, No. C-SF-15-15, Cook) was introduced 4 mm into the femoral artery through this muscular branch and remained in the artery for 1 minute. After the wire was removed, the muscular branch was ligated with 7.0 silk suture. Blood flow through the femoral artery was restored by releasing the surgical thread. The skin incision was closed with silk suture. Mice were then gavaged with 0.5 mg MZ suspended in PBS (n = 5) or PBS (n = 6) vehicle control, twice daily for 4 weeks.

### Histological and Morphometric Analyses

At the end of the protocol, mice were perfused with saline and fixed using formalin with a 25 gauge needle, inserted into the left ventricle, at a rate of 1 mL/min. The injured femoral artery was excised, embedded in paraffin blocks, sectioned (5 µm) and stained for elastin. For quantitation, 3 cross sections taken every 600 µm were selected from each artery. The images were analyzed using ImageJ software (NIH, Bethesda, MD). Intimal (I) and medial (M) areas were measured and the ratio of I/M was calculated.

### Immunofluorescence and Immunohistochemistry Staining

For cell proliferation and migration assays, MOVAS cells were fixed with cold methanol and stained for microtubules with a monoclonal anti-β-tubulin−Cy3 antibody (1∶100, Sigma, St. Louis, MO) and for nucleus with DAPI (Roche, Indianapolis, IN). For filamentous actin, cells were fixed with 4% formaldehyde and stained with Alexa Fluor® 488 Phalloidin (1∶40, Life Technologies Corporation, NY). For dead cell staining, cells were stained with 0.04% trypan blue (MP Biomedicals, LLC) for 10 minutes. For apoptotic cell staining, cells were stained with Image-iT LIVE Red Caspase Detection Kits (Life Technologies Corporation, NY) or TdT In Situ Apoptosis Detection Kit (R&D Systems, MN) following manufacturer’s instructions. For femoral artery sections vascular smooth muscle cells were detected with a smooth muscle cell α-actin monoclonal antibody (1∶1000, Cedarlane Laboratories Limited, Burlington, NC). Endothelial cells were detected with a CD31 antibody (1∶50, Abcam, Cambridge, MA). Elastin was detected with the Accustain Elastic Stain kit (Sigma, St. Louis, MO). Cells from 3 cross sections taken every 600 µm were stained with PCNA antibody (1∶50, Santa Cruz biotechnology, Inc.) to assess proliferation. Apoptotic cells were stained with TdT In Situ Apoptosis Detection Kit (R&D Systems, MN) following manufacturer’s instructions. The results were expressed as percentage of total intimal cells.

### Statistical Analysis

Values are expressed as mean ± SD. The statistical significance of differences between two groups was determined by the student 2-tailed *t* test. For multiple comparisons, results were analyzed using two-way ANOVA, followed by Bonferroni post-test analysis. *P*<0.05 was considered significant.

## Results

### Effect of MZ on VSMC Proliferation

**Figure 1:**
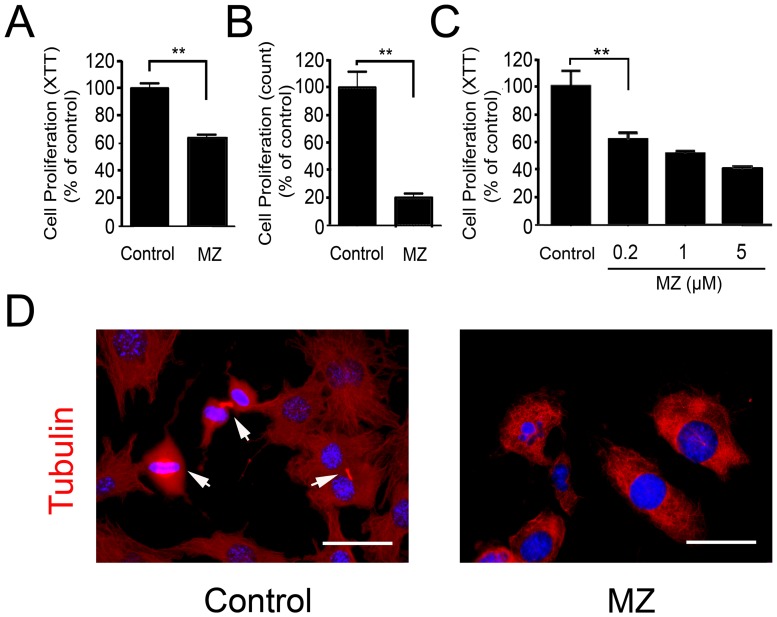
Effect of MZ on MOVAS cell proliferation. **A. MOVAS cell proliferation by XTT.** MOVAS cells were seeded in 96-well plate and incubated with 1 µM MZ for 48 hours. Cell proliferation was measured with an XTT proliferation kit and expressed as percentage of control group treated with DMSO. **B. MOVAS cell proliferation by direct counting.** MOVAS cells were seeded in 96-well plate and incubated with 1 µM MZ for 48 hours. MOVAS cells were detached and counted manually. Cell proliferation was expressed as percentage of control group treated with DMSO. **C. Dose effect of MZ on MOVAS cell proliferation.** MOVAS cells were seeded in 96-well plate and incubated with MZ at indicated concentration for 48 hours. Cell proliferation was measured with an XTT proliferation kit and expressed as percentage of control group treated with DMSO. **D. Tubulin staining.** MOVAS cells undergoing proliferation were immmunostained for tubulin (red) and nuclei (blue). Arrows point to cells undergoing different stage of cell division. Scale bar: 50 µm. **P<0.01.

Incubation of MZ (1 µM) with MOVAS cells inhibited cellular proliferation compared to control cells treated with vehicle (Fig. 1). To exclude the possibility that changes in metabolic status of the cells might affect the XTT assay result (Fig. 1A), the cells were detached and counted directly (Fig. 1B). Both results demonstrated an inhibitory effect of MZ on MOVAS cellular proliferation. MZ inhibited the MOVAS cell proliferation in a dose dependant manner (Fig. 1C).

To determine if the effects of MZ on MOVAS cell proliferation were associated with changes in the cellular distribution of microtubules, β-tubulin was stained in MOVAS cells at the end of the proliferation assay. Cells of various stages of cell division were abundant in vehicle-treated but not MZ-treated cells (Fig. 1D) in the proliferation assay.

### Effect of MZ on VSMC Migration

**Figure 2:**
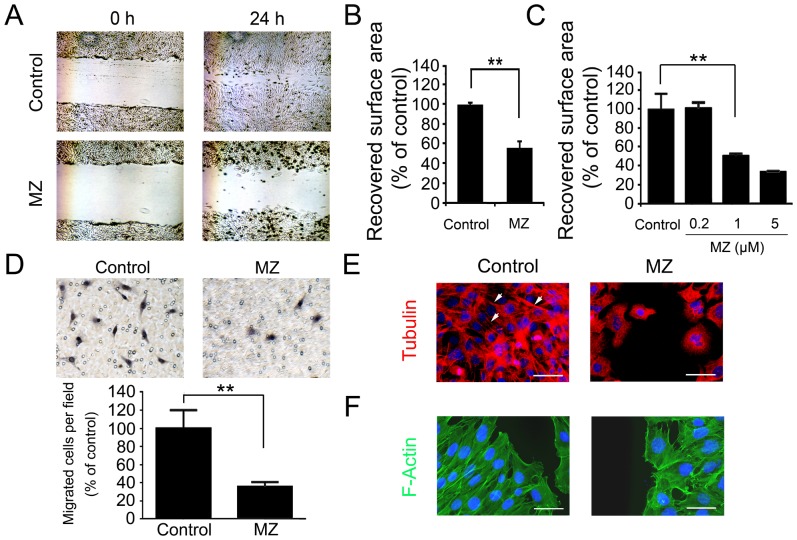
Effect of MZ on MOVAS cell migration. **A. MOVAS cell migration.** Scratch injury was performed on a confluent cell monolayer. Cells were imaged at 0 and 24 hours after injury. **B. Quantification of cell migration in wound healing assays after MZ treatment for 24 hours.** Data are presented as percentages of the recovered scratch area relative to untreated control cells. **C. Dose effect of MZ in wound healing assay.** Effect of different MZ doses on cell migration. **D. Transwell migration assay. The upper panels are representative images. The lower panel is the quantification result. E. Tubulin staining.** MOVAS cells undergoing migration were immmunostained for tubulin (red) and nuclei (blue). Arrows point to polarized microtubules. **F. Filamentous actin (F-actin) staining.** MOVAS cells undergoing migration were immmunostained for filamentous actin (green) and nuclei (blue). Scale bar: 50 µm. **P<0.01.

To assess the capacity of MZ to inhibit MOVAS cell migration, an *in vitro* model of cell injury was used. In this wound healing assay, recovery of the wounded area was measured 24 hours following the scratch. Compared to vehicle-treated cells, MZ markedly inhibited MOVAS cell migration into the wounded area (Fig. 2A, B). MZ inhibited MOVAS cell migration in a dose dependant manner and 1 µM was the minimum effective dose (Fig. 2C).

To distinguish the inhibitory effect of MZ on proliferation and migration in the wound healing assay, the transwell migration assay was also conducted. MZ also showed inhibitory effects in this migration assay (Fig. 2D).

To determine if the effects of MZ on MOVAS cell proliferation were associated with changes in the cellular distribution of microtubules, β-tubulin was stained in MOVAS cells at the end of the migration assay. Microtubules were oriented towards the direction of cell migration in vehicle-treated cells, while MZ-treated cells displayed nearly absent microtubule polarization (Fig. 2E).

To examine the effects of MZ treatment on other components of the cytoskeleton, filamentous actin (F-actin) were stained at the end of the migration assay. Actin appeared to be present in similar quantities in the MZ treated cells, however, the cellular distribution was different than control treated cells, reflecting the lack of polarity (Fig. 2F).

### Effect of MZ on VSMC Apoptosis

**Figure 3:**
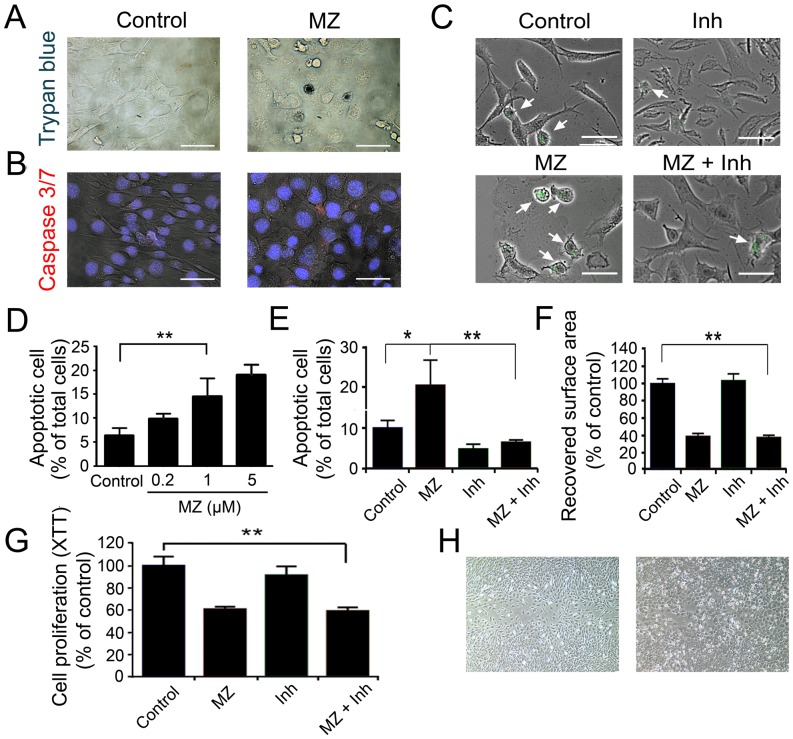
Effect of MZ on VSMC Apoptosis. **A. Dead cell staining.** Dead cells were stained with trypan blue 24 hours after injury. **B. Apoptosis cell staining.** Cells undergoing apoptosis were stained 24 hours after injury. Arrow points to active caspase 3/7 positive cell. **C. TUNEL staining.** Arrow points to apoptotic cells 24 hours after injury. **D. Dose effect of MZ on apoptosis at indicated concentration by TUNEL staining.** Data are presented as percentage of total cells. **E.** **Quantification of apoptotic cells treated with MZ or apoptosis inhibitor (Inh) in wound healing assay by TUNEL staining.** Data are presented as percentages of the total cells. **F.** **Quantification of cell migration in wound healing assay after MZ and apoptosis inhibitor (Inh) treatment for 24 hours.** Data are presented as percentages of the recovered scratch area relative to untreated control cells. **G.** **Quantification of cell proliferation after MZ and apoptosis inhibitor (Inh) treatment for 48 hours.** Cell proliferation was measured with an XTT proliferation kit and expressed as percentage of control group treated with DMSO. **H. Migrated cells treated with MZ.** 24 hours after the injury in migration assay (left), cells were treated with 1 µM MZ for another 24 hours (right). Scale bar: 50 µm. *P<0.05, **P<0.01.

As MZ has been reported to cause cellular apoptosis [12], this effect was studied in the cell migration assay and proliferation assay. At the end of cell migration assay, cells were stained with trypan blue, a vital stain for dead cells. In the scratched area, where cellular migration had occurred, there were more dead cells in the MZ-treated plate compared with the vehicle- treated plate (Fig. 3A). To determine the contribution of apoptosis to cell death, cells were stained with a caspase detection kit in which active caspase-3 and -7 were stained red. There were more apoptotic cells in the MZ-treated plate compared with vehicle-treated plate (16.25±2.58% vs 4.53±1.09%, p<0.01) (Fig. 3B). TUNEL staining also showed that MZ induced cell apoptosis in a dose dependent manner (Fig. 3D). To investigate whether the anti-migration effect of MZ was the result of apoptosis, cells were treated with 50 µM Caspase-3/7 Inhibitor I. The result of TUNEL staining showed that caspase-3/7 Inhibitor I significantly inhibited the cell apoptosis induced by MZ (Fig. 3C, E). However, cell migration was still inhibited (Fig. 3F). Similarly, caspase-3/7 Inhibitor I did not reverse the inhibitory effect of MZ on MOVAS cell proliferation (Fig. 3G).

To determine whether MZ would cause regression of VSMCs that have already migrated in response to injury, we tested the effects of MZ treatment following reconstitution of the scratch injury. 24 hours after the scratch injury, cells were treated with MZ and analyzed 24 hours later. After treatment, cell orientation and polarization was altered, however MZ treatment did not cause regression of migrated cells (Fig. 3H).

### Effect of MZ on Neointima Formation

**Figure 4:**
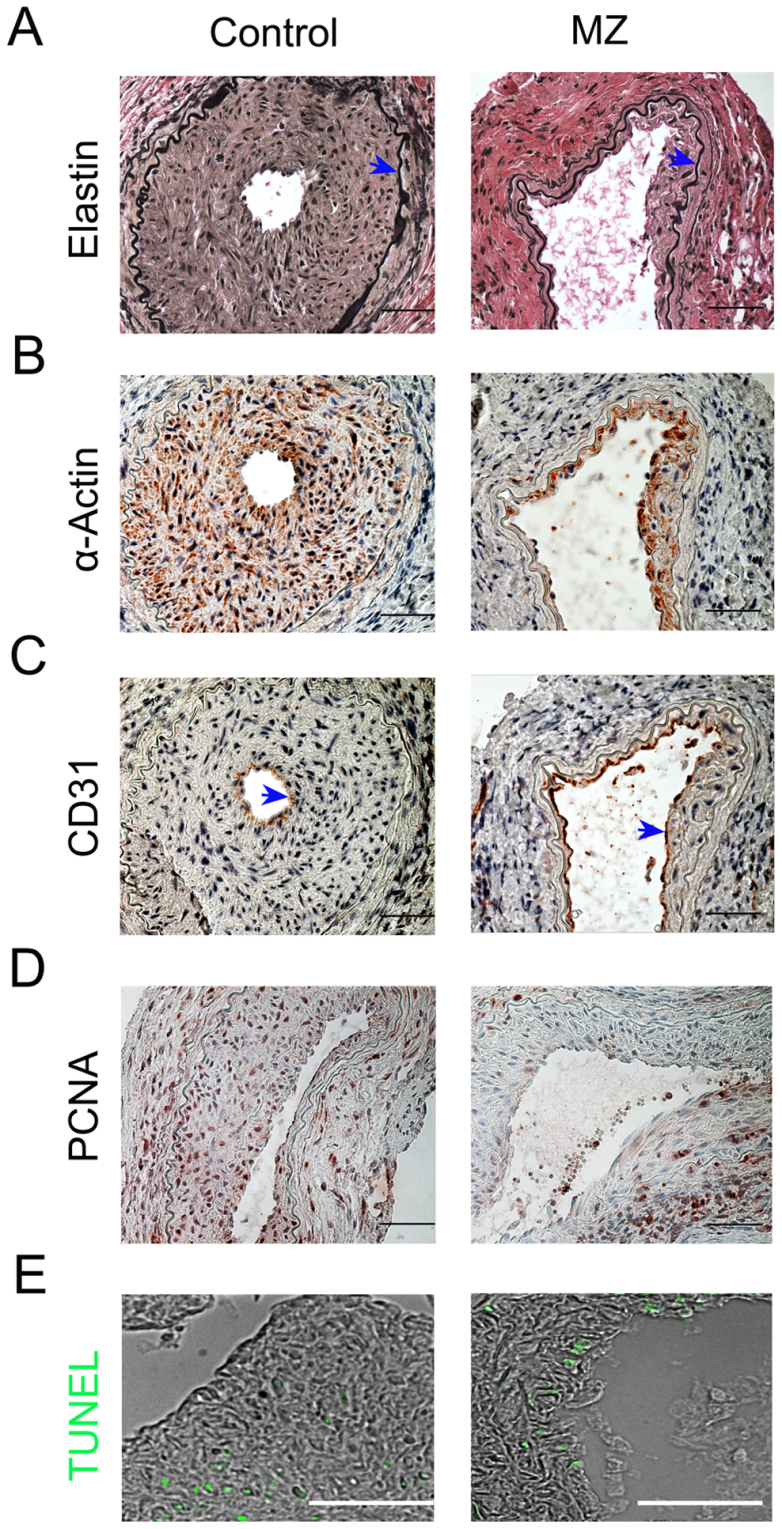
Effect of MZ on intimal hyperplasia following femoral artery wire injury. **A. Sections stained for elastin.** The area of intima and media was analyzed with ImagJ software. Arrows point to internal elastic lamina. **B. VSMC α-actin immunostaining.** Vascular smooth muscle cells (brown) were detected with α- SMC actin antibody. **C. CD 31 immunostaining.** Endothelial cells (brown) were detected with CD31 antibody. Arrows point to endothelium. **D. PCNA immunostaining.** Proliferating cells (brown) were detected with PCNA antibody. **E. TUNEL staining.** Apoptotic cells (green) were detected with TUNEL assay. Scale bar: 50 µm.

To test the *in vivo* capacity of MZ to inhibit the pathological accumulation of vascular smooth muscle cell-rich neointima, a murine model of femoral arterial injury was used. Four weeks following wire-induced femoral artery injury, the average femoral artery neointimal area in MZ-treated mice was significantly reduced compared with control mice (Fig. 4A and Table 1). There were no significant differences in the medial area between control and MZ-treated mice and the ratio of intima to media was reduced in mice treated with MZ (Table 1). The neointimal area was comprised predominantly of VSMCs in both groups of mice (Fig. 4B). Both groups demonstrated re-endothelialization as evidenced by a layer of CD31-staining cells adjacent to the lumen (Fig. 4C). Since a reported side effect of MZ is leukopenia [13], white blood cell counts were measured and found to be similar between the 2 groups of mice (Table 1). The number of cells undergoing proliferation were significantly reduced in MZ-treated mice compared to the control group (17.76±9.48% vs 31.03±10.41%, P = 0.001, Fig. 4D). However, MZ treatment did not cause more apoptosis than the control group (4.71±3.33% vs 3.8±2.12%, P = 0.61, Fig. 4E).

**Table 1:** Statistical results of femoral artery wire injury assay.

	Control	MZ	P value
Intimal area (I) (×10^3^ µm^2^)	32.8±4.7	21.1±6.5	0.02
Medial area (M) (×10^3^ µm^2^)	3.6±2.8	2.5±1.4	0.36
Intimal area/Medial area (I/M)	2.24±0.44	1.45±0.38	0.01
White Blood Cells (×10^3^/µl)	8.85±2.62	9.73±1.92	0.45

Results expressed as mean ± SD.

## Discussion

MZ is a member of the benzimidazole class of drugs and is used to treat parasitic infestations by worms including pinworms, roundworms, tapeworms, hookworms, and whipworms [6]. It is typically given 100 mg twice per day for 3 days but has been given up to several weeks [14]. The mechanism of action is thought to be interference with polymerization of tubulin to form microtubules [7], [8]. This leads to glucodepletion in the parasites due to destroyed extant cytoplasmic microtubes in their intestinal cells. Drugs that interfere with tubulin polymerization have also been found to be efficacious against proliferation of malignant cells. For example, MZ and other benzimidazole carbamate drugs have recently been tested in cancer studies and shown to suppress the growth of tumor cells *in vitro* and *in vivo* [15]–[17].

The present study demonstrates that MZ can also inhibit proliferation and migration of vascular smooth muscle cells. This is associated with disruption of microtubule organization and secondary changes in the distribution of other cytoskeleton components such as filamentous actin. MZ treatment likely prevented the formation of the mitotic spindle apparatus which inhibited cell division. Although apoptosis induced by MZ may contribute to inhibitory effects, this does not appear to be a major determinant in this model since an apoptosis inhibitor did not reverse the inhibitory effect of MZ. Importantly, MZ treatment was effective in reducing neointima formation following arterial injury. This effect was mediated by inhibition of vascular smooth muscle cell proliferation and migration at the site of injury and did not appear to impair re-endothelialization in vivo. This therapy could therefore be useful for treating vascular diseases associated with pathologic vascular smooth muscle cell proliferation. MZ is particularly attractive because of the low risk of side effects, even with long term treatment in humans [18], [19]. No apparent side effects were observed in this study.

We suspect based on the mechanism of action that this drug will be particularly effective against proliferating/migrating cells and would therefore only be useful if given shortly after the acute vascular injury, similar to other anti-restenosis therapies. Although MZ has been shown to induce apoptosis [12] and we also observed an increased rate of apoptosis in MZ-treated cells following the *in vitro* scratch injury, this was not sufficient to induce regression of migrated cells. Consistently, MZ treatment did not cause regression of the smooth muscle cells that had migrated in response to the scratch injury. This supports the concept that MZ will be effective if smooth muscle cells are in a proliferative or migrating phase.

Coronary stenting is widely utilized for the treatment of obstructive atherosclerotic coronary disease in the US [20]. Despite the widespread use of drug-eluting stents restenosis remains a pervasive problem occurring anywhere from 3–20% of patients with drug-eluting stent [21], depending on stent and patient characteristics. While dual antiplatelet therapy with aspirin and thienopyridines reduces stent thrombosis [22], drugs available to prevent recurrent restenosis are limited. Cilastazol has been shown to reduce target vessel revascularization in some studies [23] but not others [24]. Cilastazol also has antiplatelet effects which may theoretically increase bleeding risk when added to other antiplatelet agents. Other safe and effective options to treat restenosis are needed, especially in patients who experience recurrent instent restenosis.

The present study provides a potentially novel and safe strategy to target vascular diseases associated with pathologic vascular smooth muscle cell proliferation.
